# POLICY SERIES: A SECURE RETIREMENT FOR OLDER WOMEN

**DOI:** 10.1093/geroni/igad104.0419

**Published:** 2023-12-21

**Authors:** Brian Lindberg, Cindy Hounsell

## Abstract

This session will address policy development related to women and their financial well-being in later life. Panelists will discuss the recently passed SECURE 2.0, the latest programs providing financial literacy to older adults, healthcare workforce related issues, and the role of the Consumer Financial Protection Bureau is protecting older adults from fraud and elder exploitation.

